# Birth prevalence of genital anomalies among males conceived by intracytoplasmic sperm injection cycles: A cross-sectional study

**DOI:** 10.18502/ijrm.v21i1.12666

**Published:** 2023-02-08

**Authors:** Fatemeh Aliani, Zahra Haghshenas, Ahmad Vosough Dizaj, Arezoo Arabipoor, Samira Vesali, Mahnaz Ashrafi

**Affiliations:** ^1^Department of Pediatrics, Faculty of Medicine, Tehran University of Medical Sciences, Tehran, Iran.; ^2^Department of Reproductive Imaging, Reproductive Biomedicine Research Center, Royan Institute for Reproductive Biomedicine, ACECR, Tehran, Iran.; ^3^Department of Endocrinology and Female Infertility, Reproductive Biomedicine Research Center, Royan Institute for Reproductive Biomedicine, ACECR, Tehran, Iran.; ^4^Reproductive Epidemiology Research Center, Royan Institute for Reproductive Biomedicine, ACECR, Tehran, Iran.; ^5^Shahid Akbarabadi Clinical Research Development Unit (ShACRDU), Iran University of Medical Science (IUMS), Tehran, Iran.

**Keywords:** Cryptorchidism, Hypospadias, Microinjections, Prevalence, Reproductive techniques, Urogenital abnormalities.

## Abstract

**Background:**

Several studies have been conducted worldwide to evaluate the prevalence and relative risks of congenital anomalies associated with assisted reproductive technology cycles; however, there is limited data in Iran.

**Objective:**

To investigate male genital anomalies among live births from assisted reproductive technology.

**Materials and Methods:**

This cross-sectional study was conducted on children born after intracytoplasmic sperm injection (ICSI) at Royan Institute, Tehran, Iran from April 2013-December 2015. The prevalence of male genitalia disorders that included hypospadias, epispadias, cryptorchidism, micropenis, and vanishing testis were reported. The relationship between the cause of infertility and type of embryo transfer (fresh or frozen), gestational age at birth (term or preterm), and birth weight with these male genitalia anomalies were evaluated.

**Results:**

In total, 4409 pregnant women were followed after their ICSI cycles to evaluate genitalia anomalies in their children. Out of 5608 live births, 2614 (46.61%) newborns were male, of which 14 cases (0.54%) had genital anomalies. The prevalence of various anomalies were cryptorchidism (0.34%), hypospadias (0.038%), micropenis (0.038%), vanishing testis (0.038%), and epispadias (0.077%). No relationship was found between the cause of infertility, type of embryo transfer (fresh or frozen), gestational age at birth (term or preterm), and male genital malformation (p = 0.33, p = 0.66, and p = 0.62, respectively).

**Conclusion:**

The prevalence of each male genital anomaly after the ICSI cycle was rare and less than 0.5%; however, no significant infertility-related factor was observed with these anomalies.

## 1. Introduction

During the past 4 decades, assisted reproductive technology (ART) has been used as a standard worldwide medical practice to achieve pregnancy. ART is responsible for an estimated 1-5.9% of conceptions in some developed countries (1). In vitro fertilization (IVF) and intracytoplasmic sperm injection (ICSI) are the 2 main ART modalities. Because sperm and oocytes are manipulated outside the body, there are concerns regarding embryos derived from these invasive procedures (2). The findings of several studies and meta-analyses indicate that ART is associated with an increased risk of congenital anomalies (1-5). Results of previous studies have suggested an association between ART and some specific cardiac birth defects (6, 7), as well as some noncardiac defects such as cleft lip with or without cleft palate (8); hypospadias (9, 10), neural tube defects (11), esophageal, anorectal (8, 12), genitourinary defects (13), and large intestinal atresias (12). However, the magnitude of these associations and the spectrum of the involved defects remains unknown (14).

Presumed reasons to explain the higher risk of congenital malformations (CM) in ART infants include underlying subfertility, medications for ovulation induction, micromanipulation in ICSI and/or IVF, as well as increase in multiple gestations and prematurity (14). More CM have been observed in ICSI as a more invasive treatment method in cases where sperm could not pass through the natural route and physiologic deletion of abnormal sperms does not occur (oligospermia) (15). Similar interventions applied in IVF include the gonadotropin stimulator, oocyte aspiration, and culture media, which likely raise the incidence of CM (15). Some studies have investigated whether there is a higher risk of CM in offspring conceived by ICSI than IVF. However, they reported controversial results (9, 15-17). It was hypothesized that congenital genitourinary malformations, such as hypospadias, are more frequent after ICSI than after IVF. Moreover, they stated that there is a lack of robust data due to the rarity of these conditions (9). Hence, it was concluded that the increased prevalence of congenital genitourinary malformations was observed in singletons born after ART; however, more studies are warranted for confirmation (17). Only one study in Iran evaluated congenital anomalies and could not find a significant relationship between the type of infertility treatments and CM; however, the rate of CM after the ICSI cycles (11.7%) was slightly higher than after IVF cycles (5.9%) (15).

The developmental effect of ART on infants remains an important subject that needs further monitoring and investigation. This cross-sectional study with the retrospective design was done to evaluate the prevalence rate of congenital anomalies, particularly male genital malformations, in offspring conceived by ICSI at Royan Institute.

## 2. Materials and Methods

This cross-sectional study was performed at Royan Institute, Tehran, Iran from April 2013-December 2015. After registering the ICSI cycles in Hakim's software system at Royan Institute, data regarding pregnancies were investigated, and all live births were monitored and followed retrospectively. The ICSI cycles and embryo transfer procedure were performed with standard protocols at Royan Institute. We collected the data regarding the cause of infertility and type of embryo transfer from participants' records. The characteristics of newborns were also collected and recorded from the follow-up clinic. Gestational age was determined as 14 days before oocyte pick-up until delivery. To exclude outliers, only children with gestational ages between 22 and 44 wk and birth weight between 400 and 7000 gr were included. Preterm birth was considered as delivery before 37 completed weeks of gestation. Low-birth weight (LBW) was defined as 2500 gr and very LBW as 1500 gr (18).

All children were assessed at birth by neonatologists and the premature infants were reevaluated at the expected date of delivery. The diagnosis of the genital anomaly was determined on the basis of the clinical examination at the expected date of delivery. Sonography was performed if the anomaly was not diagnosed by a physical exam. A diagnosis of hypospadias was made as a failure of fusion of the urethra was observed, and the urethral meatus was ectopically located. Cryptorchidism could not be diagnosed if the testis was in the inguinal canal or not palpable. Testis in a high scrotal position was not considered to be cryptorchidism (19). Micropenis was described as a penis 2.5 standard deviations (SD) smaller than the mean for the child's age and race. It was diagnosed by observing a stretched penis length of less than 1.9 centimeters at birth. Testicular regression syndrome or vanishing testis is reported to be due to the subsequent atrophy and disappearance in the fetal life of an initially normal testis, and its diagnosis is confirmed by surgery (20). Epispadias was defined by observing the opening of the urethra in the back of the penis (19).

### Ethical considerations

The study was approved by the Institutional Review Boards and the Ethical Committees of Royan Institute, Tehran, Iran (Code: IR.ACECR.ROYAN.REC.1397.207). The patients' file information has been used while maintaining the confidentiality of names, and oral consent has been obtained from all participants.**

### Statistical analysis 

Data were analyzed using the Statistical Package for the Social Sciences 20.0 (SPSS; SPSS Inc., Chicago, IL, USA). The Kolmogorov-Smirnov test was used to evaluate the normality of continuous variables. Normally distributed continuous variables are presented as mean. Comparison of means for dichotomous variables was performed by the independent sample *t* test, and the Chi-square test was used to examine the relationship between categorical variables. A p-value < 0.05 was considered to be statistically significant.

## 3. Results

In this study, 4409 infertile couples with successful pregnancies after ICSI cycles were investigated to examine the male urogenital anomalies in their newborns. The mean age of fathers and mothers was 35.58 ± 5.64 yr and 30.86 ± 4.92 yr. Out of 4409 pregnancies, 3202 pregnancies (72.6%) were single, 1109 pregnancies (25.2%) twin, and 98 pregnancies (2.2%) triple. In total, the all-out number of live births were 5608. Of the 4409 couples studied, 1762 couples (39.9%) had female infertility, 2194 couples (49.8%) had male infertility, and 453 couples (10.3%) had both male and female infertility factors. The mean gestational age at delivery was 36.3 ± 3.2 wk. Of 5608 live births, 2152 (38.4%) were preterm and 3456 (61.6%) had normal weight. Of 5608 live births, 2614 newborns were male (46.61%), and the remaining (2994 newborns) were female. The demographic and clinical characteristics of the participating couples are shown in table I.

Out of 2614 male newborns, only 14 (0.54%) had urogenital anomalies. As seen in table II, the highest prevalence of genitalia anomalies were related to cryptorchidism [9 (0.34%)]. The lowest prevalence of genitalia anomalies were related to hypospadias, micropenis, and vanishing testis [1 (0.038%)].

Table III shows the prevalence of genitalia anomalies based on gestational age in male newborns. The highest prevalence of genitalia anomalies was related to preterm ( < 37 wk) newborns (0.98%). The most common anomaly was cryptorchidism (7 cases from 10 anomalies). The prevalence of genitalia anomalies in male term newborns ( > 37 wk) was 0.25%. The total prevalence of genitalia anomalies by gestational age in male newborns was 0.54%. The Chi-square test showed that the relationship between the prevalence of male genitalia anomalies and the gestational age was not statistically significant (p = 0.62).

Table IV shows the relationship between the cause of infertility and the type of embryo transfer and male genital anomalies. With fresh embryo transfer, the prevalence of genital anomalies was 0.71%. The Chi-square test showed that the relationship between the prevalence of genitalia anomalies and the type of embryo transfer was not statistically significant (p = 0.33). The Chi-square test demonstrated that the relationship between the prevalence of male genitalia anomalies and the causes of infertility was not statistically significant (p = 0.66). Out of 1582 male infants, with male infertility caused by their father, only 351 were diagnosed with azoospermia. In the following, out of 14 infants with male genitalia anomalies, 6 infants had a history of mild or moderate male infertility without an azoospermia diagnosis or surgery for sperm retrieval in their father (Table IV). As a result, no significant link was discovered between azoospermia diagnosis and male genital anomalies in newborns following ICSI cycles.

**Table 1:** Demographic and clinical characteristics of the 4409 infertile couples and their newborn


**Variables**		
Fathers' age (yr)*		35.5 ± 5.6
Mothers' age (yr)*		30.8 ± 4.9
Gestational age at childbirth (wk)*		36.3 ± 3.2
Weight at birth (gr)**		
	**< 1500**	433 (7.7)
	**1500-2499**	1646 (29.4)
	**2500-4000**	3456 (61.6)
	**> 4000**	73 (1.3)
Newborn status**		
	**Term**	2152 (38.4)
	**Preterm**	3456 (61.6)
	**Twin**	1109 (25.2)
	**Triple**	98 (2.2)
Cause of infertility**		
	**Female factor **	1762 (39.9)
	**Male factor**	2194 (49.8)
	**Both factors**	453 (10.3)
*Data presented as Mean ± SD. **Data presented as n (%)		

**Table 2:** Prevalence of various genitalia anomalies in male newborns


**Genitalia anomalies**	**Frequency (%)**	**Prevalence (%)**
**Cryptorchidism**	9 (64.3)	0.34
**Micropenis**	1 (7.1)	0.038
**Hypospadias**	1 (7.1)	0.038
**Vanishing testis**	1 (7.1)	0.038
**Epispadias**	2 (14.3)	0.077
**Total**	14 (100)	0.54

**Table 3:** Prevalence of genitalia anomalies in male newborns based on gestational age


	**Male genitalia anomalies**					
**Gestational**
	**age at birth**	**Live births** **(n)**	**Cryptorchidism**	**Vanishing testis**	**Micropenis**	**Epispadias**	**Hypospadias**	**Prevalence** **(%)**	**P-value**
**Preterm**	1020		7 (70)	1 (10)	0 (0%)	2 (20)	0 (0)			0.98	
**Term**	1594	2 (50)	0 (0)	1 (25)	0 (0)	1 (25)	0.25	0.62
Data presented as n (%). Chi-square test								

**Table 4:** The relationship between genitalia anomalies in male newborns after ICSI cycles with the cause of infertility and type of embryo transfer


		**Male genitalia anomalies**					
**Variable**		**No. live birth**	**Cryptorchidism**	**Vanishing testis**	**Micropenis**	**Epispadias**	**Hypospadias**	**Prevalence (%)**	**P-value**
**Type of embryo transfer**									
	**Freeze**	1073	2 (66.6)	1 (33.3)	0 (0)	0 (0)	0 (0)	0.28	
	**Fresh**	1541	7 (63.6)	0 (0)	1 (9.1)	2 (18.2)	1 (9.1)	0.71	0.33
**Cause of infertility**									
	**Female**	1007	2 (33.3)	0 (0)	2 (33.3)	1 (16.7)	1 (16.7)	0.59	
	**Male**	1358	5 (71.4)	1 (14.3)	0 (0)	1 (14.3)	0 (0)	0.53	
	**Both**	249	1 (100)	0 (0)	0 (0)	0 (0)	0 (0)	0.40	0.66
Data presented as n (%). Chi-square test. ICSI: Intracytoplasmic sperm injection									

## 4. Discussion

Epidemiological research on the distribution of various male genitalia malformation in children conceived by ART was the first fundamental step in the current study. In many parts of the world, extensive studies have been performed. Recently, in a meta-analysis study, a significant association between congenital abnormalities and ART cycles which imposes a tremendous burden on global health was reported (2). Despite these extensive studies, there is limited research on male and female genitalia malformation in Iran due to the lack of integrated electronic information of individuals' medical records. We chose to study genitalia anomalies because the genitalia system is the target of most hormonal drugs used in treatment cycles. In addition, the underlying causes of infertility are maternal and/or paternal reproductive system disorders.

In the present study, various genitalia anomalies had the following prevalences: cryptorchidism (0.34%), hypospadias (0.038%), micropenis (0.038%), vanishing testis (0.038%), and epispadias (0.077%). Due to our limitations, these children were almost entirely examined by pediatricians at the hospital's birth center. The information collected was obtained from the parents via telephone and by assessment of medical records.

In the current study, the prevalence of cryptorchidism was 0.16% (9 out of 5608 live births) and 0.34% in boys. The prevalence of this disease was 0.2% in preterm neonates and 0.09% in term infants, which is approximately 20 times higher in premature infants and is consistent with the physiopathology description of this disease. The prevalence of cryptorchidism was 4.5% in live births from natural pregnancies, approximately 30% in premature infants, and 3.4% in term newborns. The difference in the prevalence of these 2 categories is that the normal time of testicular decline is between 28 and 32 wk of gestation. Although the cause of the testicle decline and its main mechanism is still unknown, it is undoubtedly affected by 2 hormonal changes, androgens and Müllerian-inhibiting substance (21). In another study in Yazd, the prevalence of cryptorchidism in preterm and mature infants was reported as 29.5% and 3.27%, respectively (22). Mozafari Kermani and colleagues (15) studied the children who resulted from IVF or ICSI and reported a 0.5% prevalence of cryptorchidism. Similarly, a systematic review and meta-analysis determined that the prevalence of cryptorchidism in children born by ICSI was 0.53%, and concluded that there was no significant difference compared to the general population regarding this congenital anomaly (9). We found similar results with previous studies; however, a recent meta-analysis in China concluded that ART was correlated with an increased risk of congenital urinary tract malformation in offspring, especially cryptorchidism (1.83 times more) (17).

The prevalence of hypospadias was reported in 3-5 cases in 1000 natural live births (0.3-0.5%). The prevalence of hypospadias in Asia was reported to be 69 in 10,000 (0.69%) (23). Other research estimated the incidence of hypospadias as 0.38% in those born by ICSI, which did not significantly increase in the general population (9). In agreement with previous studies, the prevalence of hypospadias in the present study was 0.017%, one in 5608 cases of total births and 0.038% in male births. The average birth weight in the present study was 2890.3 gr, and the rates of LBW and premature infants were 34.4% and 61.4%, respectively. It seems that the cryptorchidism and hypospadias in children conceived by ART are due to LBW, and prematurity and male factor infertility do not correlate with an increase in the prevalence of these diseases in neonates. However, in a recent mate-analysis research reported that ART was correlated with an increased risk of hypospadias in offspring (1.87 times more) (17).

The prevalence of epispadias at birth for the whole spectrum is reported at 1 in 10,000, which ranges from 1 in 30,000 (0.003%) for classical bladder exstrophy to 1 in 200,000 for exstrophy of the cloaca, with an overall more significant proportion of affected males (24). However, in the current study, the prevalence of this anomaly was 0.035% in total live births and 0.077% in males. Compared to the mentioned prevalence, the rate of epispadias in live births after ICSI was 10 times more than reported previously in live births from natural conception. Unfortunately, there is no accurate epidemiologic study on the prevalence of this congenital anomaly in natural live births in Iran to compare. However, these reports may indicate the need for further studies on the association of ART and this anomaly and examine other types of extrophy in these children. This finding was consistent with the results of Zwink and colleagues who suggested that ART treatments such as IVF and ICSI are associated with an increased risk of the exstrophy-epispadias complex. However, it is unclear whether they are caused by ART therapy per se or based on the infertility etiology or parents' characteristics (25).

In our study, the incidence of micropenis was 0.017%. Elsewhere, in northeastern Brazil incidence of micropenis (18 cases among 2710 newborns) was reported as 0.66% (26). Unfortunately, this prevalence is not available in the natural live births in Iran for comparison and further discussion.

The only strengthof thisstudyis the first report of the prevalence of male genitalia malformations after ICSI cycles in Iran. However, this study has some limitations that should be mentioned. One limitation was that in our institute, the treatment cycles were defined as IVF/ICSI cycles and ICSI cycles. Since in the IVF/ICSI cycles, it was not determined whether the fetus was the result of IVF or ICSI procedure, and the IVF cycle alone is not a routine at Royan Institute; however, a non-ICSI group for comparison was not available. In addition, it was not possible to collect the clinical characteristics and information of babies from natural pregnancies for the researchers. Therefore, we compared our results with the prevalence of these anomalies in natural pregnancies reported in the published scientific articles.

## 5. Conclusion

The results of the study indicated that the prevalence of each male urogenital anomalies including cryptorchidism, hypospadias, micropenis, and vanishing after the ICSI cycle was rare and less than 0.5%, and almost similar to those in infants from natural pregnancies which reported in Iranian scientific literature; however, we had no control group for better comparison. Therefore, a prospective study with a control group should confirm the present results.

##  Conflict of Interest 

The authors declare that there is no conflict of interest.
